# Antioxidant and Microbiota Characteristics Across Different Mucosal Sites of Rice Flower Carp (*Procypris merus*)

**DOI:** 10.3390/microorganisms13122673

**Published:** 2025-11-24

**Authors:** Huige Ren, Yutu Tang, Jingyi Du, Zihao Xu, Xiao Peng, Ye Qian, Zihe Guo, Chanxia Qin, Shihao Li, Sikai Huang, Yonggang Mo, Chengrui Huang, Weihao Ou

**Affiliations:** Key Laboratory of Aquatic Healthy Breeding and Nutrition Regulation of Guangxi Universities, College of Animal Science and Technology, Guangxi University, Nanning 530004, China

**Keywords:** fish, gill, skin, intestine, oxidative stress, microecology

## Abstract

Antioxidant and microbiota in different mucosal sites of fish play important roles. However, relevant research is lacking for rice flower carp (*Procypris merus*). This study investigated antioxidant and microbiota characteristics across different mucosal sites (gill, skin, and intestine) of this fish. Antioxidant analysis revealed the following: catalase activity followed gill > intestine > skin (*p* < 0.05); total superoxide dismutase activity showed intestine > gill > skin (*p* < 0.05); malondialdehyde level in the gill significantly exceeded the skin and intestine (*p* < 0.05); and, superoxide anion level ranked gill > intestine > skin (*p* < 0.05). The intestinal microbiota had the significantly lowest α-diversity (*p* < 0.05). Across different mucosal sites, LEfSe analysis revealed differentially abundant genera, and microbial functional prediction (BugBase) showed significant differences in Forms Biofilms, Potentially Pathogenic, Stress Tolerant, and Gram-Positive (*p* < 0.05). Correlation analysis between differentially abundant genera and antioxidant indicators revealed multiple significant positive correlations (*p* < 0.05) but no significant negative correlations (*p* > 0.05) in the gill; only two significant negative correlations (*p* < 0.05) and no significant positive correlations (*p* > 0.05) in the skin; and no significant correlations (*p* > 0.05) in the intestine. Collectively, these findings might contribute to the microecological regulation of rice flower carp.

## 1. Introduction

With the rapid growth of the global population, the increasing demand for animal protein has driven the aquaculture industry toward intensification [1]. According to a Food and Agriculture Organization (FAO) report, global aquaculture production steadily increased from 6 million tons in 1975 to 126 million tons in 2021 [2]. However, as aquaculture scales expand, challenges such as malnutrition, weakened disease resistance, and declining flesh quality have emerged, constraining the industry’s healthy development [3]. Previous studies have indicated that high stocking densities and poor water quality increase environmental stressors, leading to oxidative stress in farmed animals [4]. Under oxidative stress, cellular damage caused by reactive oxygen species (ROS) accumulation reduces antioxidant and immune capacity [5]. During farming, this leads to widespread disease outbreaks and mortality—particularly severe in juvenile fish—significantly impeding sustainable aquaculture development [4,6].

Fish mucosal tissues—such as gill, skin, and intestine—serve as the primary barrier against the external environment, playing vital roles in immunity, defense, and physiological regulation [7]. Direct exposure to aquatic surroundings makes these tissues vulnerable to oxidative stress and pathogen infection, causing immunosuppression, growth retardation, and disease outbreaks [8,9,10,11]. Therefore, investigating antioxidant and microbiota characteristics across different mucosal tissues is essential for enhancing disease resistance and farming efficiency in aquatic animals.

The antioxidant system is an essential component of the mucosal defense mechanisms in fish [12], serving as the first line of defense against oxidative stress [13]. It includes enzymatic antioxidants such as superoxide dismutase (SOD), catalase (CAT), and glutathione peroxidase (GPx), as well as non-enzymatic antioxidants like glutathione and vitamin E [14]. Antioxidant enzymes including GPx, SOD, and CAT scavenge ROS and play crucial roles in preventing oxidative damage [15]. Malondialdehyde (MDA), an end product of lipid peroxidation, serves as a valuable indicator for assessing oxidative stress status and antioxidant levels [16]. Furthermore, diverse microbial communities inhabit mucosal surfaces, where they contribute significantly to nutrient absorption, immune enhancement, and pathogen suppression [17]. The stability and diversity of these microbiota directly influence mucosal function and health [17]. A growing body of evidence suggests that mucosal commensals can bolster the host’s antioxidant status through tissue-specific mechanisms. In the intestine, certain probiotics are known to directly enhance the activity of host antioxidant enzymes [18,19,20]. On the skin, resident commensals can competitively exclude pathogens, thereby reducing inflammatory responses and indirectly mitigating local ROS production [21,22,23]. Although direct evidence is more limited in the gill, its complex microbiota is also postulated to mitigate oxidative stress from waterborne pollutants via immunomodulation or microbial metabolism [23,24]. However, the precise regulatory networks and the extent of these microbiota–antioxidant interactions across different mucosal niches remain to be fully elucidated.

Rice flower carp (*Procypris merus*; genus *Procypris*, family Cyprinidae) is commonly and widely farmed in rice-fish farming systems [25]. In these systems, the fish primarily consumes rice flowers that fall into the water from the rice plants [26]. Rice flower carp is distinguished by its soft, edible intermuscular bones and the absence of the muddy odor typically found in other freshwater fish [27]. Additionally, its high protein content has made it a highly valued aquatic product among consumers [28]. The cultivation of rice flower carp has a rich history of over a thousand years in China, where it has significantly contributed to increasing farmer incomes and promoting agricultural development [25]. Despite its importance, scientific research on rice flower carp is still in its infancy. Most existing studies focus on the immune response under environmental stressors [25,28]. A systematic comparative investigation of the antioxidant and microbiota characteristics across its different mucosal sites, which are critical interfaces for health in its aquatic environment, is entirely lacking. Furthermore, to the best of our knowledge, no study has ever characterized the microbiota of its gill and skin. Therefore, this study aims to explore the antioxidant and microbiota characteristics in the gill, skin, and intestine of rice flower carp, investigate their interrelationships, and provide a scientific basis for improving mucosal antioxidant capacity and microbiota, thereby promoting its sustainable farming and disease control.

## 2. Materials and Methods

### 2.1. Sample Collection

The experiment was conducted at a commercial aquaculture farm in Nanning, China. A total of 15,000 juvenile rice flower carp (average body weight: 5.20 ± 0.04 g) were evenly distributed into 30 net cages (5 m × 5 m × 2.5 m) within a single 0.8-ha earthen pond. The same pond was selected for the experiment to minimize the impact of environmental heterogeneity, which is well-established in fish physiological and microbiological research [29,30,31]. The fish were fed a commercial diet (Zhanjiang Haida Feed Co., Ltd., Zhanjiang, China) twice daily (08:00 and 17:00) at 3–5% of their body weight for three months. The commercial diet contained ≥35.0% crude protein, ≥4% crude fat, ≥8.5% crude fiber, and ≥1.65% lysine. Water quality parameters were maintained within optimal ranges: temperature 25–28 °C, dissolved oxygen ≥ 5.0 mg L^−1^, ammonia-N ≤ 0.1 mg L^−1^, and nitrite ≤ 0.01 mg L^−1^. Throughout the experiment, no significant disease outbreaks, with a survival rate of 92.4 ± 1.3%. At the end of the trial, nine healthy fish (average weight: 51.06 ± 0.86 g) were randomly selected from three randomly chosen net cages (3 fish per cage; each fish was treated as an independent biological replicate; n = 9). The fish were anesthetized with MS-222 (100 mg/L; Sigma-Aldrich, St. Louis, MO, USA). Using sterile instruments, skin samples from above the lateral line on the left dorsal side, the first gill filament samples from the outer to inner part of the left gill arch, and hindgut samples were collected. Samples were separately placed in sterile enzyme-free centrifuge tubes for antioxidant and microbiota analyses. Following flash-freezing in liquid nitrogen, samples were stored in a −80 °C ultra-low temperature freezer.

### 2.2. Measurement of Antioxidant Indicators

Approximately 0.1 g of tissue was homogenized in ice-cold sterile physiological saline solution (*w*/*v*, 1:9) using an Ultra-Turrax homogenizer (Tekmar Co., Cincinnati, OH, USA) and centrifuged at 4000 r/min at 4 °C for 10 min to obtain the supernatant for further analysis. Protein concentration, MDA level, CAT activity, and total superoxide dismutase (T-SOD) activity in gill, skin, and intestine samples were measured using kits from Nanjing Jiancheng Bioengineering Institute (Nanjing, China) (Item Nos.: A045-2-2, A003-1-2, A007-1-1, and A001-3-2, respectively). Superoxide anion (O_2_^−^) levels were determined using a kit from Beijing Solarbio Science & Technology Co., Ltd. (Beijing, China) (Item No.: BC1295). All procedures strictly followed the manufacturers’ protocols. All absorbance readings were performed using a FlexA-200 microplate reader (Allsheng Inc., Hangzhou, China). The specific activities or concentrations were calculated according to the manufacturers’ protocols and were normalized to the protein content of the supernatant, expressed as units or nanomoles per milligram of protein (U/mg prot or nmol/mg prot).

### 2.3. Genomic DNA Extraction and 16S rRNA Gene Amplicon Sequencing

Total microbial DNA was extracted from mucosal samples using the CTAB method. Amplification targeted the V3–V4 region of the 16S rRNA gene with primers (F: CCTAYGGGRBGCASCAG; R: GGACTACNNGGGTATCTAAT). The PCR mixture contained 15 μL Phusion^®^ High-Fidelity PCR Master Mix (New England Biolabs, Inc., Ipswich, UK), 0.2 μM primers, and 10 ng genomic DNA template. The PCR program was set as: initial denaturation at 98 °C for 1 min; 30 cycles of 98 °C for 10 s, 50 °C for 30 s, and 72 °C for 30 s; final extension at 72 °C for 5 min. PCR products were purified with magnetic beads, pooled in equal amounts according to their concentrations, mixed thoroughly, and the target bands were recovered. The purified PCR products were used for library construction. Qualified libraries, verified by Qubit and qPCR quantification, were sequenced on the Illumina NovaSeq6000 platform (PE250) at Novogene Co., Ltd. (Beijing, China). The raw data of 16S rRNA gene amplicon sequencing had been uploaded to the Sequence Read Archive (SRA) with the accession number PRJNA1297164.

### 2.4. 16S rRNA Gene Amplicon Sequencing Data Processing

The sequencing data were demultiplexed based on barcode sequences and PCR amplification primer sequences. After removing barcodes and primer sequences, reads from each sample were assembled using FLASH (v1.2.11, http://ccb.jhu.edu/software/FLASH/ (accessed on 25 February 2025)) to generate Raw Tags. Use the Cutadapt software (Version 3.3) to match the reverse primer sequence and trim off the remaining part of the sequence to prevent it from interfering with subsequent analysis. Subsequently, Raw Tags were stringently filtered using fastp software (Version 0.23.1) to obtain high-quality Clean Tags. The Clean Tags sequences were compared against the Silva database (SILVA database (16S/18S), https://www.arb-silva.de/; accessed on 25 February 2025) to detect and remove chimeric sequences, yielding the final Effective Tags. These Effective Tags were denoised via the DADA2 module in QIIME2 software (Version QIIME2-202202) to derive final Amplicon Sequence Variants (ASVs) and feature tables.

### 2.5. Bioinformatics Analysis

Taxonomic annotation was performed using QIIME2 software, and rapid multiple sequence alignment established phylogenetic relationships for all ASVs. Data were rarefied to the minimum sample depth for subsequent α- and β-diversity analyses. The α-diversity indices (Chao1, Shannon, Simpson, Pielou_e) were calculated with QIIME2. Venn diagram was generated using the VennDiagram function in R. Principal Coordinates Analysis (PCoA) was computed and visualized via the ade4 and ggplot2 packages in R (Version 4.0.3). Histograms of relative abundance distribution were plotted in Perl using SVG functions, depicting the top 10 species at both phylum and genus levels for each sample. LEfSe (LDA Effect Size) identified differentially abundant genera across different mucosal sites. Microbiota functional profiling was predicted using BugBase (https://bugbase.cs.umn.edu/index.html; accessed on 25 March 2025). Finally, Pearson correlation clustering heatmaps analyzed associations between differentially abundant genera and antioxidant indicators in the gill, skin, and intestine of rice flower carp.

### 2.6. Data Processing and Statistical Analysis

Data was tested for normality and variance homogeneity using the Shapiro–Wilk W goodness of fit test and the Bartlett test, respectively. These data was subjected to one-way analysis of variance (ANOVA) using SPSS 22.0 for Windows. When overall differences were significant (*p* < 0.05), differences among means were evaluated by Duncan’s multiple comparison test. Data are presented as the mean ± SEM.

## 3. Results

### 3.1. Antioxidant Characteristics Across Different Mucosal Sites of Rice Flower Carp

Antioxidant indicators were measured in three primary mucosal tissues: gill (HG), skin (HS), and intestine (HI). Results revealed significant differences in antioxidant profiles across different mucosal sites (Table 1). CAT activity ranked HG > HI > HS (*p* < 0.05). T-SOD activity followed HI > HG > HS (*p* < 0.05). MDA level in HG was significantly higher than that in HS and HI (*p* < 0.05). O_2_^−^ level exhibited HG > HI > HS (*p* < 0.05).

### 3.2. Microbiota Characteristics Across Different Mucosal Sites of Rice Flower Carp

**Figure 1:**
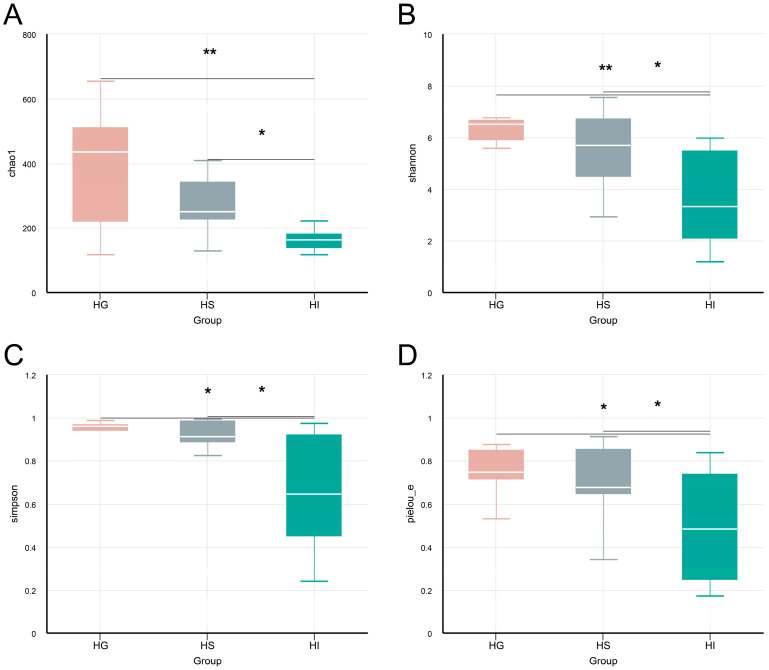
The α-diversity analysis of the mucosal microbiota of rice flower carp. (**A**) Chao1 index; (**B**) Shannon index; (**C**) Simpson index; (**D**) Pielou_e index. HG: Gill; HS: Skin; HI: Intestine. * indicates *p* < 0.05, ** indicates *p* < 0.01.

The α-diversity of the mucosal microbiota differed significantly among tissues (Figure 1). Results showed no significant differences between the HG and HS microbiota in any α-diversity index (*p* > 0.05; Figure 1A–D). The HI microbiota exhibited significantly lower Chao1, Shannon, Simpson, and Pielou_e indices than the HG and HS microbiota (*p* < 0.05; Figure 1A–D).

**Figure 2:**
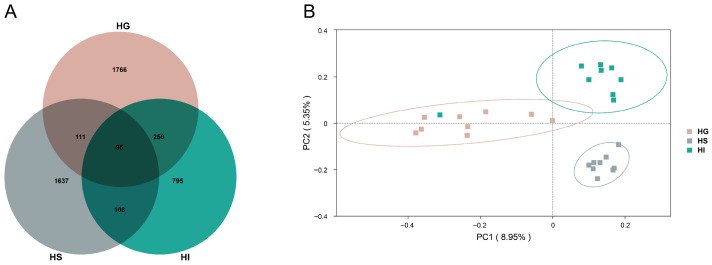
The ASV distribution and the β-diversity characteristics of the mucosal microbiota of rice flower carp. (**A**) Venn diagram (ASV level); (**B**) PCoA plot. HG: Gill; HS: Skin; HI: Intestine.

Analysis of ASV numbers and the β-diversity further confirmed distinct community structures (Figure 2). The Venn diagram revealed 95 shared ASVs among the three mucosal sites, with the HG, HS, and HI containing 1766, 1637, and 795 unique ASVs, respectively (Figure 2A). Additionally, the PCoA plot of the β-diversity analysis demonstrated distinct clustering of microbial communities from the HG, HS, and HI in the ordination space (Figure 2B).

**Figure 3:**
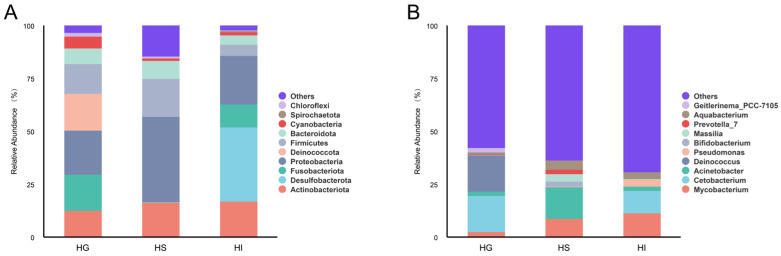
Top 10 phyla and genera by relative abundance of the mucosal microbiota of rice flower carp. (**A**) Top 10 phyla; (**B**) Top 10 genera. HG: Gill; HS: Skin; HI: Intestine.

To further elucidate the primary composition of the mucosal microbiota of rice flower carp, this study statistically analyzed relative abundance at phylum and genus levels (Figure 3). Results showed the top 10 phyla by relative abundance as: Actinobacteriota, Desulfobacterota, Fusobacteriota, Proteobacteria, Deinococcota, Firmicutes, Bacteroidota, Cyanobacteria, Spirochaetota, and Chloroflexi (Figure 3A). At the genus level, the top 10 genera by relative abundance were: *Mycobacterium*, *Cetobacterium*, *Acinetobacter*, *Deinococcus*, *Pseudomonas*, *Bifidobacterium*, *Massilia*, *Prevotella*_7, *Aquabacterium* and *Geitlerinema*_PCC-7105 (Figure 3B).

**Figure 4:**
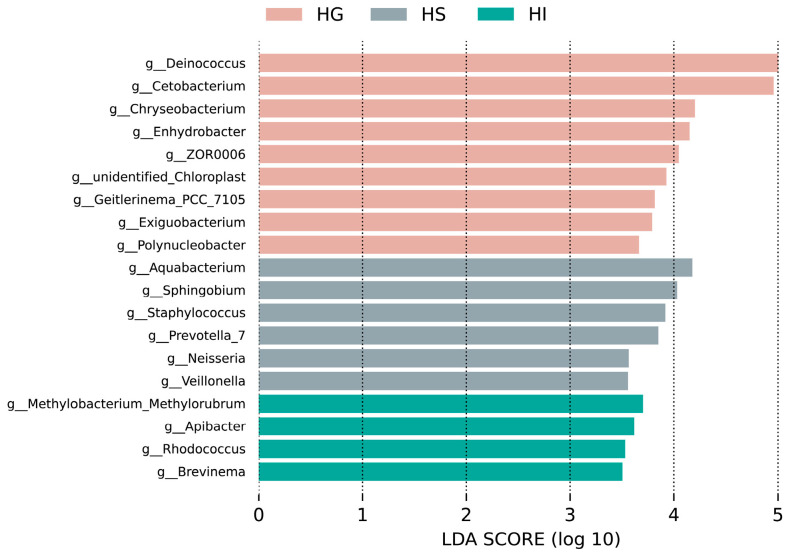
LEfSe analysis of differentially abundant genera of the mucosal microbiota of rice flower carp (LDA > 3.5). HG: Gill; HS: Skin; HI: Intestine.

LEfSe analysis identified genera that were significantly enriched in one mucosal site (Figure 4). Results indicated that significantly differentially abundant genera in the HG included *Deinococcus*, *Cetobacterium*, *Chryseobacterium*, *Enhydrobacter*, ZOR006, unidentified_Chloroplast, *Geitlerinema*_PCC_7105, *Exiguobacterium*, and *Polynucleobacter*; in the HS: *Aquabacterium*, *Sphingobium*, *Staphylococcus*, *Prevotella*_7, *Neisseria*, and *Veillonella*; in the HI: *Methylobacterium_Methylorubrum*, *Apiabacter*, *Rhodococcus*, and *Brevinema*.

**Figure 5:**
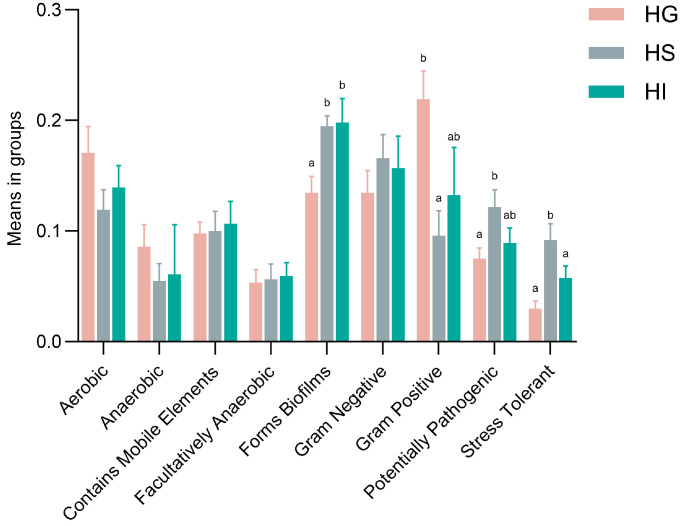
Functional prediction (BugBase) of the mucosal microbiota of rice flower carp. HG: Gill; HS: Skin; HI: Intestine. Different lowercase letters indicate significant differences (*p* < 0.05).

Functional prediction using BugBase showed significant phenotypic differences (Figure 5). Compared with the HG microbiota, Forms Biofilms significantly increased in the HS and HI microbiota (*p* < 0.05). Compared with the HG microbiota, the HS microbiota exhibited significantly reduced Gram-Positive (*p* < 0.05), while showing increased Potentially Pathogenic and Stress Tolerant phenotypes (*p* < 0.05). Furthermore, Stress Tolerant phenotype in the HS microbiota was significantly higher than that in the HI microbiota (*p* < 0.05).

### 3.3. Correlation Analysis Between Differentially Abundant Genera and Antioxidant Indicators Across Different Mucosal Sites of Rice Flower Carp

**Figure 6:**
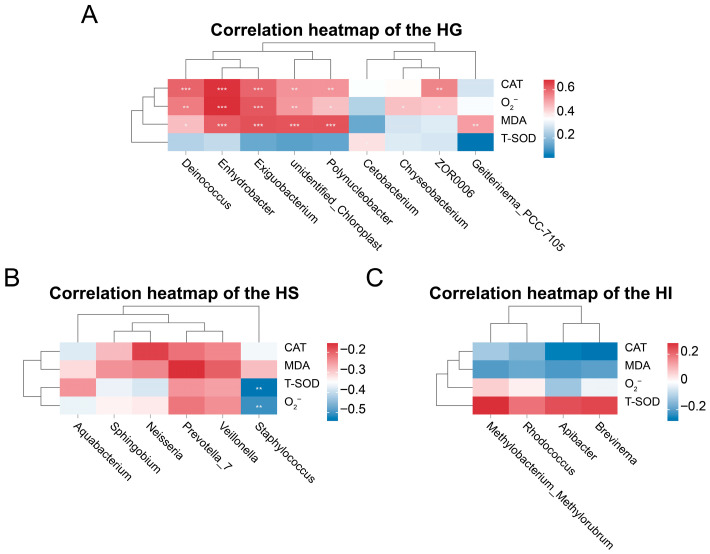
Correlation analysis between differentially abundant genera and antioxidant indicators across different mucosal sites of rice flower carp. (**A**) HG: Gill; (**B**) HS: Skin; (**C**) HI: Intestine. * indicates *p* < 0.05, ** indicates *p* < 0.01, *** indicates *p* < 0.001.

Correlation analysis highlighted site-specific relationships between differentially abundant genera and antioxidant indicators (Figure 6). Among differentially abundant genera in the HG (Figure 6A), the relative abundances of *Deinococcus*, *Enhydrobacter*, *Exiguobacterium*, unidentified_Chloroplast, and *Polynucleobacter* showed significantly positive correlations with CAT activity, MDA levels, and O_2_^−^ levels (*p* < 0.05), the relative abundance of *Cetobacterium* exhibited a significantly positive correlation with T-SOD activity (*p* < 0.05), the relative abundance of *Chryseobacterium* showed a significantly positive correlation with O_2_^−^ levels (*p* < 0.05), the relative abundance of ZOR006 demonstrated significantly positive correlations with both CAT activity and O_2_^−^ levels (*p* < 0.05), and the relative abundance of *Geitlerinema*_PCC_7105 showed a significantly positive correlation with MDA levels (*p* < 0.05). In the HS (Figure 6B), among differentially abundant genera, the relative abundance of *Staphylococcus* exhibited significantly negative correlations with O_2_^−^ levels and T-SOD activity (*p* < 0.05). In the HI (Figure 6C), differentially abundant genera *Methylobacterium*_*Methylorubrum*, *Apiabacter*, *Rhodococcus*, and *Brevinema* showed no significant correlations with CAT activity, MDA levels, O_2_^−^ levels, or T-SOD activity (*p* > 0.05).

## 4. Discussion

The antioxidant system serves as a critical defense line against oxidative stress in fish mucosa, playing a pivotal role in maintaining cellular redox homeostasis and preventing tissue damage [13]. Among these components, antioxidant enzymes such as CAT and SOD effectively scavenge ROS in vivo, constituting essential defenses against oxidative stress [32]. Meanwhile, MDA and O_2_^−^ levels reflect the extent of lipid peroxidation and free radical clearance efficiency in organisms [33,34]. This study revealed significant differences in antioxidant capacity across distinct mucosal tissues of rice flower carp, confirming tissue-specific defense mechanisms against oxidative stress in fish [35]. The gill exhibited the significantly highest CAT activity and the significantly second-highest T-SOD activity after the intestine, while simultaneously showing significantly higher MDA and O_2_^−^ levels than both the skin and intestine. This phenomenon might relate to the gills’ persistent exposure to the aquatic environment and their involvement in physiological functions, including gas exchange, nitrogen excretion, and osmoregulation, rendering them more susceptible to ROS attack [36]. Similar patterns have been observed in other fish species. For example, in common carp (*Cyprinus carpio*) exposed to environmental stressors, gills consistently exhibited the highest antioxidant enzyme activities, yet this was accompanied by an increase in oxidative damage markers, indicating a high vulnerability to oxidative challenge [37]. Consequently, gills face elevated oxidative damage risks and demonstrate greater reliance on efficient antioxidant enzyme systems [35]. The skin showed the lowest CAT activity, T-SOD activity, MDA levels, and O_2_^−^ levels, likely because humoral immune factors (e.g., antimicrobial peptides, lysozymes, proteases) in its mucus layer sufficiently counteract most external threats, thereby reducing local ROS generation pressure and consequently lowering induction and activity demands on the antioxidant enzyme system [38]. This is consistent with findings in Atlantic salmon (*Salmo salar* L.), where the skin mucosa demonstrated a stronger reliance on non-enzymatic barriers and immune components rather than enzymatic antioxidants under normal conditions [38,39]. The intestine demonstrated the significantly highest T-SOD activity and relatively low MDA and O_2_^−^ levels, indicating robust redox regulatory capacity. This might be closely linked to intestinal microecological modulation, as studies suggest certain commensal bacteria act as antigens to stimulate antioxidant enzyme secretion, thereby effectively alleviating oxidative stress [40,41]. Such intestine-specific antioxidant enhancement has also been reported in rainbow trout (*Oncorhynchus mykiss*), where the gut mucosa exhibited a uniquely high capacity for SOD upregulation in response to probiotic supplementation, highlighting the importance of microbial interactions in shaping intestinal antioxidant responses [42]. These results underscored structural and functional specificity across different mucosal tissues.

Mucosal tissues such as the gill, skin, and intestine in fish provide specific colonization environments for symbiotic microbial communities due to their unique structures and physiological functions [43]. The stability of mucosal microecology also contributes to maintaining host health [44]. Previous studies have indicated mucosa-specific microbial assemblages across different sites [43,45,46,47]. The α-diversity and β-diversity of mucosal microbiota serve as key indicators for assessing microbiota stability [48]. Current mucosal microbiota research on rice flower carp is limited to the intestine [49,50]. In this study, the intestinal microbiota exhibited significantly lower Chao1, Shannon, Simpson, and Pielou_e indices than the gill and skin microbiota, indicating reduced species richness and evenness, while the gill and skin microbiota demonstrated greater diversity and stability. Aligning with patterns observed in other fish species such as large yellow croaker (*Larimichthys crocea*) and sofie fish (*Parachondrostoma toxostoma*), where external mucosal surfaces generally hosted richer and more diverse microbial communities than the gut [43,45]. This difference might relate to the degree of exposure to microorganisms in the surrounding aquatic environment [43]. Additionally, the relatively anaerobic environment in the intestine might restrict microbial diversification, favoring a community dominated by a few taxa, as also reported in Nile tilapia (*Oreochromis niloticus*) [51,52]. Notably, no significant differences in microbial α-diversity were detected between the gill and skin, possibly due to similarities in their mucosal niche characteristics and environmental exposure, a phenomenon similarly documented in Pacific chub mackerel (*Scomber japonicus*) [53]. Furthermore, this study revealed that dominant bacterial phyla and genera across different mucosal sites of rice flower carp resembled those reported on mucosal sites of other freshwater fish (e.g., *Cyprinus carpio*, *Oreochromis niloticus* and crucian carp (*Carassius auratus gibelio*) [52,54,55].

Based on LEfSe analysis, this study identified differentially abundant genera across different mucosal sites, revealing unique microecological structures and functional preferences. The gill exhibited significant enrichment of microbiota, balancing antioxidant repair and organic matter metabolism. *Deinococcus* is renowned for its superior antioxidant enzyme activity and DNA repair mechanisms [56], and its enrichment might reflect the gill’s demand or stress adaptation to high-ROS environments. *Cetobacterium*, a core commensal in freshwater fish intestine, synthesizes vitamin B_12_ and short-chain fatty acids (e.g., acetate, butyrate) [57], and its cross-site presence might support local energy metabolism. Concurrently, enrichment of opportunistic pathogens like *Chryseobacterium* and *Enhydrobacter* might result from rice flower carp’s exposure to complex aquatic environments, suggesting potential microbial barrier vulnerability to invasion under high-pressure niches [58,59,60]. In the skin, taxa including *Aquabacterium* and *Staphylococcus* were significantly enriched. *Aquabacterium*, an aerobic gram-negative bacterium common in freshwater biofilms [61,62], likely contributes to organic substrate decomposition on this oxygen-rich surface. *Staphylococcus*, often a resident on fish skin, might act as an indirect pathogen that facilitates colonization of other harmful microbes, as similarly observed in *Oncorhynchus mykiss* skin microbiota [63,64]. Intestinal enrichments featured *Methylobacterium*_*Methylorubrum*, *Apiabacter*, *Rhodococcus*, and *Brevinema*, reflecting functional adaptation to an anaerobic, nutrient-rich milieu. The methylotrophic metabolism of *Methylobacterium*_*Methylorubrum* can yield antioxidant intermediates [65,66], whereas *Apiabacter*—common in insect guts—participates in amino acid and monosaccharide metabolism [67,68]. *Rhodococcus*, with its broad organic degradation capacity, might assist in dietary substrate breakdown, a role also noted in catla (*Catla catla*) gut microbiota [69,70]. By contrast, *Brevinema* is an opportunistic pathogen in red hybrid tilapia (*Oreochromis* spp.) and was associated with elevated mortality during co-infection with viruses [71]. Together, these mucosal microbes appeared to form an intricate and niche-adapted network that might support host physiology through substrate degradation, oxidative damage repair, and immunomodulation.

Functional profiling further highlighted divergent microbial colonization strategies across mucosal niches in rice flower carp [72]. The skin microbiota displayed higher “Potentially Pathogenic” potential than the gill microbiota, along with greater “Stress Tolerant” capacity compared to both the gill and intestinal microbiota—a pattern also documented in *Larimichthys crocea* skin microbiota under fluctuating environmental conditions [43]. These traits might reflect the skin’s direct exposure to aquatic pathogens and physicochemical variabilities, necessitating enhanced defense and stress adaptation. Both the skin and intestinal microbiota demonstrated significantly stronger “Forms Biofilms” function than the gill microbiota. Increased biofilm-forming capacity facilitates microbial colonization [73]. By contrast, the gill hosted more bacteria with a “Gram Positive” phenotype, whose thick peptidoglycan layer might better withstand hydrodynamic shear and oxidative stress [74]. Collectively, mucosal microbiota functions of rice flower carp exhibited significant site-specificity, indicating high microbial adaptation to host tissue microenvironments. This functional differentiation might critically maintain local mucosal immune balance, barrier defense, and host health.

This study further revealed potential associations between differentially abundant genera and antioxidant indicators through correlation analysis. In the gill, multiple dominant genera showed significant positive correlations with key oxidative parameters, implying their hypothetical involvement in responding to local oxidative pressure. Specifically, the increased relative abundances of *Deinococcus*, *Enhydrobacter*, *Exiguobacterium*, unidentified_Chloroplast, and *Polynucleobacter* were positively correlated with the elevated CAT activity, MDA, and O_2_^−^ levels. This pattern implied that these taxa might either thrive under high oxidative stress or indirectly stimulate host antioxidant enzyme production through ROS-generating metabolic processes. A similar association has been reported in the gill microbiota of hypoxia-stressed *Larimichthys crocea*, where oxidative-tolerant bacteria were positively linked to antioxidant enzyme levels [75]. Additionally, *Cetobacterium* abundance was positively correlated with T-SOD activity, consistent with the possibility that its metabolites, such as short-chain fatty acids or vitamin B_12_, might enhance SOD-mediated superoxide clearance, as also observed in a study on largemouth bass (*Micropterus salmoides*) [76]. Conversely, *Chryseobacterium* relative abundance positively correlated with O_2_^−^ level, consistent with its potential proliferation characteristics as an opportunistic pathogen in high-ROS environments [59]. In the skin, *Staphylococcus* relative abundance showed significantly negative correlations with both O_2_^−^ level and T-SOD activity. This could indicate that *Staphylococcus* might suppress ROS accumulation by stimulating host secretion of antimicrobial peptides or expression of non-enzymatic antioxidants [38,77]. Comparable antioxidant modulation by skin commensal bacteria has been described in studies on fish skin, in which certain microbiota were shown to assist in ROS reduction via immune-mediated pathways [78]. However, the intestinal differentially abundant genera (*Methylobacterium*_*Methylorubrum*, *Apiabacter*, *Rhodococcus*, and *Brevinema*) exhibited no significant correlations with any antioxidant indicators. This outcome highlighted the complexity in redox balance regulation within the intestinal microenvironment: its homeostasis might depend more on alternative antioxidant systems (e.g., glutathione system) or unrecognized core functional microbiota. These differentially abundant intestinal genera likely primarily engaged in nutrient metabolism or immunomodulation within the intestinal ecology rather than directly mediating host oxidative stress responses. Collectively, these site-specific microbiota–antioxidant relationships suggested potential diversified strategies for maintaining redox homeostasis regulation across different mucosal sites and highlighted potential host–microbe associations in antioxidant defense, offering preliminary insights into the future targeted modulation of functional keystone bacteria to optimize mucosal health and environmental stress resilience.

Despite the novel insights provided by this study, several limitations should be acknowledged. First, all fish were sourced from a single pond to minimize the impact of environmental heterogeneity. This design means that the findings are primarily applicable to fish reared under similar conditions. Therefore, caution is warranted when generalizing these results to other environments or production systems. Second, the sample size, though sufficient for preliminary characterization, remained relatively small. Increasing the number of individuals across multiple rearing batches or environmental settings in future work would further improve statistical robustness and the generalizability of the observed patterns. Third, the functional predictions of microbiota were based on 16S rRNA gene amplicon sequencing and BugBase. While informative, these predictions were inferential and would be strengthened by multi-omics approaches—such as metagenomics, metatranscriptomics, or metabolomics—to directly elucidate microbial gene expression, metabolic pathways, and their causal relationships with host antioxidant responses. Finally, although correlation analyses suggested potential interactions between specific microbial genera and antioxidant indicators, these associations did not imply causality. Future work should include in vitro culture of isolated strains and in vivo colonization experiments to functionally validate the roles of key bacteria in modulating mucosal redox homeostasis.

## 5. Conclusions

This study demonstrated tissue-specific antioxidant and microbiota characteristics across the gill, skin, and intestine of rice flower carp. In antioxidant capacity, the gill exhibited the significantly highest CAT activity and the significantly second-highest T-SOD activity after the intestine, along with significantly higher MDA and O_2_^−^ levels than the skin and intestine; the skin showed the significantly lowest CAT activity, T-SOD activity, MDA level, and O_2_^−^ level; the intestine demonstrated the significantly highest T-SOD activity. Regarding microbiota, the intestinal microbiota displayed the lowest diversity and stability, while the gill and skin microbiota showed higher values; LEfSe analysis, functional prediction, and microbiota–antioxidant correlation analysis further identified functionally distinct microbial taxa contributing through specialized pathways. Collectively, these findings provide a foundational characterization of mucosal microecology in rice flower carp. The identified tissue-specific patterns could serve as a baseline for future studies to functionally validate the roles of key bacteria in health regulation.

## Figures and Tables

**Table 1:** Antioxidant characteristics across different mucosal sites in rice flower carp.

	HG	HS	HI
CAT (U/mg prot)	56.12 ± 1.16 ^c^	26.90 ± 0.98 ^a^	31.52 ± 1.43 ^b^
T-SOD (U/mg prot)	14.95 ± 0.81 ^b^	3.42 ± 0.63 ^a^	17.57 ± 0.61 ^c^
MDA (nmol/mg prot)	23.78 ± 2.45 ^b^	4.47 ± 0.71 ^a^	4.69 ± 0.09 ^a^
O_2_^−^ (nmol/mg prot)	319.68 ± 7.23 ^c^	65.92 ± 7.33 ^a^	176.61 ± 8.05 ^b^

Notes: HG: Gill; HS: Skin; HI: Intestine. CAT: Catalase; T-SOD: Total superoxide dismutase; MDA: Malondialdehyde; O_2_^−^: Superoxide anion. Different lowercase superscript letters within a row indicate significant differences (*p* < 0.05).

## Data Availability

The original contributions presented in this study are included in the article. Further inquiries can be directed to the corresponding author.
